# Using andragogy and instructional design to teach workshops on systematic searching in an academic library: case report

**DOI:** 10.5195/jmla.2026.2185

**Published:** 2026-01-01

**Authors:** Erin Roga

**Affiliations:** 1 e.roga@federation.edu.au, Federation University Australia, Australia

**Keywords:** Systematic review, knowledge synthesis, academic library, instructional design, searching, andragogy

## Abstract

**Background:**

Knowledge syntheses require complex searches of the literature, but many have poor quality, irreproducible search methods. Academic libraries support researchers conducting knowledge syntheses in many ways, including providing training such as workshops. However, for training to be successful, effective teaching theories and methods need to be used, such as andragogy and instructional design. These can help to develop learning strategies and experiences based on the needs of the learners.

**Case Presentation:**

At Federation University Australia Library, in response to increasing requests for support from researchers conducting knowledge syntheses, a series of workshops on systematic searching was developed using adult learning methods. We aimed to deliver quality, engaging learning experiences to researchers, and using instructional design was likely to help us meet this goal. Learning outcomes were identified, followed by developing active, collaborative learning strategies and activities. After implementation, the workshops were evaluated informally, resulting in planned changes and improvements to future offerings.

**Conclusions:**

Using andragogy and instructional design was a successful method of developing the workshops as it provided a structure to follow, and centered researcher needs. While positive feedback was received from workshop participants, there is a need to formally evaluate the learning outcomes to determine if the workshops resulted in improvements in systematic searching practices. The approach to developing the workshops can be adapted by other libraries delivering similar training on systematic searching. It is our aim that by promoting the use of effective teaching methods, the quality of search methods in knowledge syntheses will improve.

## BACKGROUND

Systematic reviews collect and synthesize evidence to provide clear answers on a research question, using a rigorous methodology to reduce bias [1]. This need for rigorous, unbiased synthesis has been adapted for a variety of aims and disciplines with at least 41 varieties of knowledge syntheses identified [2, 3]. These require complex, systematic searches of the literature to find all potential evidence [4]. However, many systematic reviews published in peer reviewed journals have significant problems with reliability and validity, with poor quality or irreproducible searches [5–7].

Libraries support researchers conducting knowledge syntheses by offering online material, consultations, training and co-authorship, and requests for support have increased over time [8–10]. Workshops have been used to meet this demand in a more sustainable manner, aiming to improve the ability of researchers to conduct high quality, reproducible searches [9]. For workshops to achieve this goal, the potential for learning needs to be maximized, which in turn requires using evidence-based teaching methods.

Unlike pedagogy, which addresses the learning needs of children, andragogy addresses the needs of adult learners such as researchers through the following principles:

- Adults need to know why they need to learn something
- Adults see themselves as autonomous and self-directed learners
- Adults use their prior life experience when learning
- Adults are ready to learn what they need for real-life situations
- Adults’ orientation to learning is contextual and problem solving
- Adults are intrinsically motivated to learn [11]

Andragogy has been successfully utilized when teaching information literacy to university students [12–14]. Learning Outcome 2.2 of ‘The Australian and New Zealand Information Literacy Framework’ states “the information literate person constructs and implements effective search strategies” [15]. As creating effective, sensitive searches when conducting knowledge syntheses requires a high degree of information literacy, it can be extrapolated that andragogy will also be successful when teaching systematic searching. To apply the principles of andragogy in practice, instructional design (ID), “deciding what methods of instruction are best for bringing about desired changes in student knowledge and skills”, is a useful model, ensuring effective teaching methods for adult learners are used [16].

Johnson-Barlow and Lehnen [17] identified 16 different ID models used in academic library instruction, with ADDIE the most frequent. ADDIE uses the following five steps:

- **Analyze** what the learning needs are likely to be
- **Design** learning strategies to meet these needs
- **Develop** activities and learning experiences
- **Implement** the learning experiences
- **Evaluate** how effective these were at meeting learning needs [18].

These steps can then be used to guide the development of library instruction and the selection of teaching methods.

### Teaching Methods

Analyzing anticipated learning needs and turning them into intended learning outcomes has been used by libraries when developing education for researchers. In their course for graduate students on systematic reviews, McGowan et al. [19] determined these from their experience conducting reviews and knowledge of the literature. Threshold concepts can also be useful to identify learning needs. These are transformative concepts which are challenging to understand, but once mastered, open new ways of thinking. They recognize that learning is an individual process, which aligns with the andragogical principle that learners build on their own unique prior experiences [11, 20]. In workshops on systematic searching, Poole [21] expected the threshold concepts to be subject headings, grey literature and search evaluation, and used these to plan learning outcomes, while recognizing that learning is an individual journey, and not all learners will reach the same outcomes from the same experiences.

Learning strategies and experiences need to be designed to meet learner needs. One such strategy is active learning, in which learners complete a task, think about it and make connections. This can aid engagement and promote deeper learning and higher order thinking [22]. However, this can be challenging to apply in online teaching. Methods such as break-out rooms, collaborative tools, discussions, and real-life tasks have been successfully used by the University of Sydney Library in online workshops on systematic searching, maintaining the engagement and interaction of in person workshops [10].

Another strategy is flipped classrooms, where students engage in online content through pre-reading or completing activities prior to attending a class, so class time can be used for active learning [23]. This strategy has been used in library workshops on systematic reviews to give learners a baseline understanding, with feedback from participants indicating the learning outcomes were met and their confidence at conducting systematic searches improved [21, 24, 25].

While adults use their prior experiences when learning, collaborative activities promote learning from peers’ experiences. In peer learning, instructors and students learn reciprocally, empowering researchers to learn from each other in a safe, supportive manner and develop a sense of independence and confidence in their abilities and expertise [26]. Collaborative activities have been used to teach systematic searching, making the content more relevant and promoting peer learning as participants supported each other to solve problems related to their own reviews [10, 24, 27].

Andragogy states that adults are oriented to learn to solve real-life problems, and reflection is a learning experience which can lead to making connections between learning and practice [28]. Reflection is part of the experiential learning cycle developed by Kolb [29], in which after an experience, the learner reflects on it, develops new ideas and theories, and applies these to practice. Reflection as meta-cognition can also support solving complex problems, as it can result in knowledge becoming integrated with what is already known [30]. It has been explicitly built into library workshops on systematic searching by Lenton and Fuller [31] and Poole [21] in which participants reported increased confidence with systematic searching and recognized areas they wished to learn more about.

## CASE PRESENTATION

Federation University Australia is a small institution in Victoria with a total of approximately 1300 FTE employees and 8700 FTE students as of December 2024, with campuses in regional towns and the capital city, Melbourne [32]. The library has eight liaison librarians, whose role is to support the teaching, learning and research in the university.

In 2020, there was a marked increase in research consultations relating to knowledge syntheses such as scoping and systematic reviews. It became evident that the researchers held many common misconceptions that have been previously identified [33, 34], for example the difference between systematic and scoping reviews, systematic searching methods, and the appropriate use of conduct and reporting guidance. To manage the volume of requests and more efficiently educate researchers, we decided to develop more comprehensive support for reviews.

The first resource created in 2022 was an online guide ‘Reviewing the literature’ [https://libguides.federation.edu.au/reviewingtheliterature], intended to provide information that could be accessed at point of need. While the guide was well used, there were still numerous requests for research consultations. This prompted us to run webinars providing information about knowledge syntheses and demonstrating search techniques. An Open Educational Resource (OER) ‘Introducing scoping and systematic reviews’ [https://oercollective.caul.edu.au/scoping-systematic-reviews/] was then developed, intended to be an interactive, easy-to-understand resource on conducting scoping and systematic reviews which both simplified and referred back to methodological and reporting guidance.

During this time, information literacy classes for undergraduates were being redesigned to include active learning and discussions to try and improve student engagement and better meet intended learning outcomes. This led us to consider how these strategies could be applied when supporting researchers conducting knowledge syntheses.

Our objectives were to provide quality learning experiences and facilitate improvements in participants’ ability to conduct reproducible and high-quality systematic searches. We determined that a series of hands-on workshops was likely to meet these goals. We also needed to design them for online delivery, as researchers are located across Victoria.

Our process began by reading examples of how other libraries delivered similar training on systematic searching [9, 10, 19, 21, 25, 27, 31]. We also explored the theory of andragogy, how using ID aids applying these theoretical principles in practice and evidence for the effectiveness of ID. We then looked at models and frameworks we could follow and determined that ADDIE fit our purpose as it supports the principles of andragogy and has been used by many other libraries [17]. The following section describes in detail the process of using the ADDIE framework.

### Analyze

The first step in designing the workshops was analyzing what the learning needs of researchers were likely to be. These were identified from challenges and misconceptions observed in research consultations, the steps taken in conducting knowledge syntheses, and feedback and observations from previous library webinars. The learning needs chosen were systematic searching techniques and appropriate use of reporting and methodological guidelines.

To meet these needs, we developed a series of five two-hour online workshops open to all staff, PhD, and Masters students pursuing research, regardless of discipline. The workshops are run by the specialist Liaison Librarian (Reviews Protocols) with a second librarian experienced in systematic searching also attending to provide additional support.

The learning needs were then turned into intended learning outcomes which stated what participants would know and be able to do at the end of each workshop (Table 1). They were limited to a maximum of three for each workshop to allow for in-depth exploration. From observing common challenges during previous webinars and research consultations, we determined that subject headings and search translation were likely to be threshold concepts for our participants.

**Table 1:** Workshop intended learning outcomes

Workshop	Intended learning outcomes
Planning the search	Creating relevant and appropriate search concepts from the review question Choosing appropriate limits and filters for the review question, and locating published search filters Using seed papers to identify relevant key words authors have used for each search concept
Developing the search	Finding relevant and comprehensive subject headings and keywords for search concepts
Putting together the search	Combining search terms correctly using wildcards, truncation, and Boolean and proximity operators
Testing and translating the search	Testing the search strategy in a database, and identifying and correcting errors Translating the search syntax, field codes and subject headings to run correctly in different databases
Extending and reporting the search	Understanding the importance of including grey literature in a review, choosing the most appropriate type, and searching for it Understanding the importance of reporting the search according to reporting guidelines and assessing the completeness of reporting in published reviews

Full lesson plans available CC BY-NC 4.0 on Open Science Framework [https://osf.io/cpqd2/].

### Design

The next stage was considering which learning strategies would address the intended learning outcomes in an online environment. We determined that active learning and a flipped classroom would be effective methods that aligned with the andragogical principles of adults as autonomous, intrinsically motivated learners focused on solving a real-life problem.

Our prior experience providing webinars on knowledge syntheses showed that delivering the content and demonstrating search techniques during the session took up a significant amount of time. We had identified a need for hands-on practice and discussion, so a flipped classroom was an appropriate strategy to achieve this. On registration, the link to ‘Introducing scoping and systematic reviews’ [https://oercollective.caul.edu.au/scoping-systematic-reviews/] was provided and participants asked to read the relevant section and complete the activities prior to the workshop. This was intended to provide an opportunity for participants to practice searching skills and have a foundation of knowledge to build on, which was briefly revised at the commencement of each workshop.

We chose the strategy of active learning as it helps relate abstract concepts to real-life situations. We applied this by developing activities about systematic searching and prompting participants to make explicit links with their own knowledge synthesis. We used a scaffolded structure in which a skill was modelled or practiced together, then small groups collaborated on a similar task, and finally the class discussed applying the skills to their own research.

### Develop

Once the strategies were decided on, the next step was to determine which activities would be effective. We focused on including group tasks and reflection in each workshop, so that participants could learn from their peers’ experiences and reflect on how what they learnt was applicable to their own knowledge synthesis.

Whole class and small group collaborative activities were developed for each learning outcome. The class activity was led by librarians as we guided participants to complete the tasks, but in the group activities we aimed to act as facilitators to enable peer learning. In the workshop on planning the search, the class looked at an example knowledge synthesis question and identified the search concepts, discussing their reasoning. They then worked in groups to identify concepts from a different example question, then discussed as a class what the concepts for their own search would be. Discussions were a crucial part of the workshops, as they empowered participants to assist each other, rather than relying on the ‘expert’ librarian to provide answers. Break-out rooms and online collaboration tools optimized engagement and interaction, and these have also been used successfully in other training for researchers [25, 27, 35].

Reflection was explicitly encouraged in our workshops by asking prompting questions to aid participants to apply learning to their own knowledge synthesis. Andragogy states that adults build on prior experiences and by asking questions such as ‘What do you know now that you didn’t before?’ and ‘Have you changed your mind about anything?’, participants were able to share their prior knowledge and misconceptions, how their thinking had changed, and how the skills would be used when developing their own search.

At the conclusion of each workshop, participants are given suggested homework to apply the knowledge and techniques learnt to their own projects.

### Implement

To implement our workshops, we developed detailed lesson plans along with supporting material [https://osf.io/cpqd2/]. The two librarians who delivered and supported the workshops met to run through the content and activities and test the technology. The workshops generally ran smoothly, with the main issues around participants accessing shared documents, lack of familiarity with online tools or programs, or problems with their technology devices.

The benefits of the flipped classroom were possibly mitigated if participants did not complete the pre-reading. Although we did not formally assess this, informal feedback within the workshops indicated most had completed at least part of it. However, we found participants still had differing skills and knowledge. While Poole [21] managed this by requiring the successful completion of a quiz prior to enrolment, we did not take this approach. While we reminded people that they would gain the most out of the workshops if they engaged with pre-reading material, having this as a requirement is at odds with the principles of andragogy [11]. Instead, we adjusted the workshops to spend more time on discussions about the topics each class found challenging. This required constant monitoring of discussions and questions to make these decisions and meet participants’ needs immediately. This was challenging in an online workshop as we could not observe facial expressions and body language, and therefore we frequently paused to ask if people had any questions.

### Evaluate

The final stage of the ADDIE framework, evaluation, is an ongoing process. Internal library feedback surveys are routinely sent to webinar and workshop participants, but in this case, we did not receive enough responses for meaningful evaluations and did not apply for ethics approval to report on the few we received. Therefore, our evaluation is informal, based on our observations of the workshops, and librarian meetings to reflect on the workshops.

Like other programs which used formal participant self-evaluation and feedback, overall, we observed improvements in confidence, planned changes to practice and satisfaction in teaching strategies [19, 21, 24, 25, 27]. In meetings to reflect on the workshops, we observed that despite the flipped classroom, participants appeared to experience a high degree of difficulty with the threshold concepts of subject headings and search translation, and for a couple, a decline in self-confidence. We also observed participants had difficulty in areas we had not anticipated, such as documenting their search development and applying the learning to their own research question.

We found that using andragogy and ID provided a clear structure for us to follow when developing the workshops and kept us focused on the needs of learners. We also found our plans could not be static. Although we planned each workshop extensively, we often needed to adapt them in the moment to meet learner needs and to revise them for future iterations based on feedback and observations. While they were a significant time commitment, we found the workshops extremely rewarding to facilitate as we learnt about the diverse areas participants were researching, and through the ongoing interaction, our professional relationships with them were strengthened.

Our evaluation has led to changes in the next workshop series. To reduce the cognitive load of two-hour online workshops, we will trial ten workshops of one hour. We developed a search log template on which we will ask participants to record their research question prior to attending, allowing them to more explicitly relate the learning to their own research and build a draft search strategy over the series. We will continue to use a flipped classroom, but as participants have varying knowledge to build on or may not have engaged with the pre-reading, we will develop a more scaffolded approach for threshold concepts in which the content is broken into smaller chunks, so that participants fully understand one part before moving on to the next. For example, subject headings will be broken up into first understanding what they are and why to use them, then how to find and select them, and finally how to add them to the search strategy.

Perhaps the most significant observation was the dip in a couple of participants’ self-confidence. While we reassured them that systematic searching is challenging and takes a lot of practice to master, this will need more consideration on how to address this. In the Information Search Process model, feelings of confusion, frustration and doubt are to be expected and common when learning to search, with reflection suggested as a strategy to manage this [36]. This indicates a need for us to encourage not only explicit reflection on the concepts, but also on the development of participants’ understanding. One possible way to achieve this is using online polls or chat to ask participants to state what they have learnt, making their learning visible to themselves and their peers.

## DISCUSSION

There have been previous reports and descriptions of library workshops for researchers conducting knowledge syntheses which mention the teaching strategies and methods used, including identifying learner needs, flipped classrooms, active learning, peer learning and reflection, [9, 10, 19, 21, 25, 27, 31]. Two reports mention that ID or adult learning principles were used to develop workshops but neither describes specific models [10, 25]. McGowan et al. describe their use of backwards design, however as their course was for credit, assignments and assessments were also part of their process [19]. This report differs in that it explicitly describes the complete process of using andragogy and ID to develop and deliver training without formally assessing learning.

Our aim was to improve researchers’ ability to conduct quality and reproducible search strategies in practice. However, a limitation of this report is our inability to determine if the workshops achieved this goal. Through observations and participants’ comments we can ascertain that in general, most felt more confident and competent, however this may not translate to real-life application of learning. These workshops are a substantial time commitment for both librarians and participants, and if they are not effective at improving searches in practice, then it is clear they need to be rethought and revised. For this reason, further research is currently underway to formally assess the effectiveness of the workshops at improving participants’ systematic searching skills by looking at future knowledge syntheses they publish.

We expect the volume of consultation requests about knowledge syntheses will continue to increase, and if the workshops are effective, they have the potential to reduce this demand. They can also be adapted to provide instruction in the increasing number of Masters and Honors courses at Federation University Australia where students are given scoping or systematic reviews as assignments. Finally, this method of using andragogy and ID, including our lesson plans on OSF [https://osf.io/cpqd2/], could be adapted or reused by other institutions and libraries which provide similar support for knowledge syntheses, taking into consideration their own unique context, culture and researcher needs.

## CONCLUSION

The well-documented phenomena of irreproducible, poor-quality searches in knowledge syntheses is a significant problem and librarians are in a position to help solve it. As requests for support are increasing, training for researchers needs to be both sustainable and effective. Using ID and models such as ADDIE can aid librarians to develop programs that provide researchers with the best opportunity for learning and although it can be a lengthy process, the potential outcomes make it worth investing the time. It is our goal that librarians are inspired by this report to use ID when designing researcher training, so that the quality and reproducibility of knowledge syntheses improve.

## Data Availability

Lesson plans associated with this article are available in the Open Science Framework [https://osf.io/cpqd2/].
